# Mirrors Can Affect Growth Rate, Blood Profile, Carcass and Meat Traits and Caecal Microbial Activity of Rabbits Reared in a “Small Group” Free-Range System

**DOI:** 10.3390/ani9090639

**Published:** 2019-09-01

**Authors:** Nadia Musco, Pietro Lombardi, Nicola Francesco Addeo, Giulia Secci, Giuliana Parisi, Maria Elena Pero, Giovanni Piccolo, Antonino Nizza, Fulvia Bovera

**Affiliations:** 1 Department of Veterinary Medicine and Animal Production, University of Napoli Federico II, Via F. Delpino 1, 80137 Naples, Italy; 2 Department of Agri-Food Production and Environmental Sciences-Section of Animal Sciences, University of Firenze, Via delle Cascine 5, 50144 Florence, Italy; 3 Department of Pathology, Anatomy and Cell Biology, Columbia University, New York, NY 10032, USA; 4 Department of Agricultural Sciences, University of Napoli Federico II, Via Università 133, 80055 Portici, Italy

**Keywords:** environmental enrichment, animals’ welfare, meat quality, dressing out, meat to bone ratio, hematological traits, serum biochemistry, short-chain fatty acids—SCFAs

## Abstract

**Simple Summary:**

The rabbit farming sector is going through a difficult period. The reduction in the consumption of rabbit meat and the increased attention paid by consumers to the welfare of farmed animals require the adoption of farming methods that are as “natural” as possible and at the same time may ensure the maintenance of good growth performance. In this sense, free-range breeding on the ground and in colonies allows the rabbits to express more natural behaviour, but it also presents some negative aspects such as decreased growth performance, higher energy expenditure of subjects due to a higher locomotion activity, and the need for a larger rearing space. Mirrors can represent a valid solution by improving the rabbits welfare and at the same time ensuring good growth performance and carcass quality traits.

**Abstract:**

The aim of this work was to propose a model of free-range raising for rabbit able to maximize the animal welfare and at the same time the productive performances through the use of mirrors. A total of 81 rabbits were allocated into free-range areas and divided into three groups (nine replicates per group): in the first group (face to face, F2F), the rabbits of each replicate could see each other. In the second group (blind) each replicate was isolated from the others; in the third group (mirrors), the replicates were divided as for the Blind group but two mirrors were placed in a corner of the perimeter. The blind group rabbits showed the lowest final weight (*p* < 0.05), while rabbits from the mirrors groups showed the best FCR and net dressing out values. The blind group showed the highest production of total short chain fatty acids, acetate (*p* < 0.05) and propionate (*p* < 0.01). The F2F rabbits showed higher levels of creatine phosphokinase and lactate dehydrogenase and lower values of blood glucose than those of the other groups, due to the higher locomotion activity. The use of mirrors can improve rabbit’s growth performance and carcass traits by lowering the rabbit’s locomotion activity in comparison to the other tested systems.

## 1. Introduction

Nowadays, rabbit breeding for meat production is going through a difficult period due to both the collapse of meat consumption and the increase of the production costs [1]. Among the different strategies aimed to boost the consumption of rabbit meat, there is the rapprochement of the consumers to this product also by proposing housing systems that are more respectful of animal welfare.

Free-range breeding on the ground and in colonies is considered one of the most natural forms of farming, since the animals can completely express their behaviour. In the extensive systems, rabbits may be kept under various conditions, with different stocking density and groups size [2], but the general tendency is to raise together the same number of animals found in the natural colonies (6–20 rabbits) and to slaughter the animals after 90 days of age, because a longer time is required to reach an adequate live weight. Despite this, the free-range farming of rabbits poses several problems under the production point of view [3]. First, when the rabbits are raised on the ground, the increase of group size negatively affects the productive performance due to unfavourable hygiene conditions [4,5]. In addition, as the age of the animals increases, the possible occurrence of aggressiveness after the 80th day of age and the existence of hierarchy seem to limit the group size [6,7,8,9]. Finally, it is important to underline that at the same stocking density, a large number of raised animals implies a large surface of the housing systems, thus potentially leading to a detrimental effect on the productive performance due to the high locomotion activity [8].

The use of mirrors would determine the effect of perceived social contact on the rabbits without increasing the need for available space or provision of olfactory contact. Previous experiments have shown that the use of mirrors can reduce the incidence of stereotypies in socially isolated horses and the heart rate of isolated heifers [10,11]. Jones and Phillips [12] found that mirrors increased behavioural complexity (expressed as the number of behaviours performed per minute) in pet rabbits, offering some advantages in terms of animal welfare. An ethological study on the use of mirrors in caged rabbits showed that the animals, either alone or grouped, preferred the cage side covered with mirrors compared to the cage side without them [13]. On the other hand, the same authors considered too high the cost of mirrors installation in the cages. For this reason, very few studies investigated the effects of mirrors on rabbit growth performance [14] and they were always performed on a low number of animals.

The aim of this work was to propose a model for the free-range raising of rabbits able to maximize the animals’ health and productive performance. To this purpose, small groups each consisting of three rabbits were raised on the ground in neighbouring areas. In the first thesis, each group can have a visual and olfactive contact with the others, and in the second, each group was isolated from the others to minimize the physical contact among and thus reduce sanitary risks. In the third thesis, low-cost mirror panels were utilised in the isolated small groups to detect possible effects of a “perceived” social contact on rabbit growth rate, blood profiles, carcass and meat traits as well as on microbial activity in the caecal content.

## 2. Materials and Methods 

### 2.1. Experimental Design, Animals and Diets

All the animals were treated according to the principles stated by the EC Directive 2010/63/UE, regarding the protection of animals used for experimental and other scientific purposes. The experimental procedures were approved by the Ethical Animal Care and Use Committee of the University of Napoli Federico II, Italy (prot. N. 2019/0058989).

**Figure 1:**
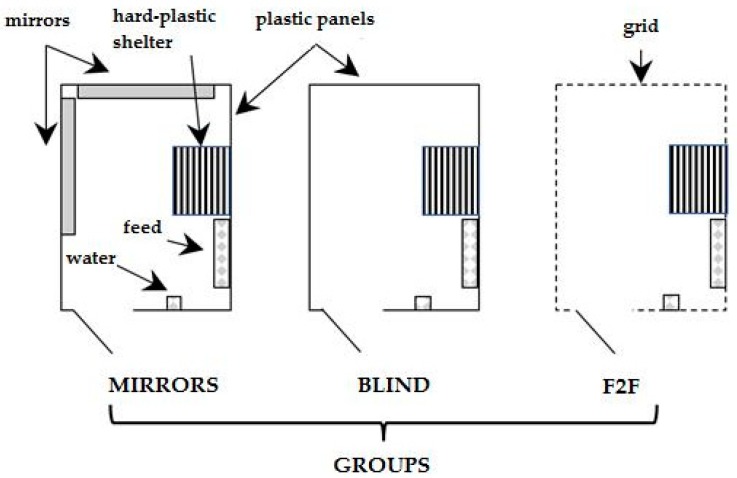
Design of the pens for Mirrors, Blind and Face to Face (F2F) groups.

The trial was carried out in a private farm in Avellino (Italy) from March 20 to May 09 2019. A total of 81, male rabbits (New Zealand White × California), weaned at 32 days of age, were moved from the bi-cellular cages into free-range areas at 45 days of age, and divided into three groups (27 rabbits, i.e., nine replicates of three rabbits per group) according to the live weight. The 3 rabbits of each replicate were from three different bi-cellular cages. In the free-range areas (2.0 × 1.5 m), the available space was 1 m^2^/head, so that each ground pen for replicate (three rabbits) was 3.0 m^2^. Each free-range area was enclosed by a 2 m high metal galvanised net with a shirt of 75 × 50 mm and a wire diameter of 2.2 mm and was protected by a shaded net deny access to predators. Three “feeding points”, containing troughs and nipples for the distribution of fresh water, were organized in each area under a plastic canopy. In the free-range area, the floor was made of compacted soil, in each pen there was a hard-plastic shelter (Long 50 × High 22.2 × Wide 40 cm) and trees, but not grass, so no additional feed was available to the animals. In the first group (face to face, F2F group), the rabbits of each replicate could see each other and could also have an olfactory contact. In the second group (blind group) each replicate was isolated from the others by blackout plastic sheets placed along the entire perimeter of the housing systems; in the third group (mirrors group), the replicates were divided as for the Blind group but two mirrors (120 × 40 cm each) were placed in a corner of the perimeter near the feeding area to form a 90 degree angle. A schema of the housing systems is reported in Figure 1. Each rabbit was identified by an ear tag.

Along the trial, the average minimum and maximum temperature registered in the area where the rabbits were kept were 8.75 °C ± 2.0 and 17.0 °C ± 2.3, respectively and the average humidity was 72.0% ± 15.8.

All the groups were fed the same commercial diet administered along the first period of the farming in cage. Feeds and water were administered ad libitum. The chemical composition and the ingredients of the diet, determined according to Association of Official Analytical Chemists (AOAC) [15], are shown in Table 1. The amount of the digestible energy (DE) has been estimated according the equation proposed by Fernandez-Carmona et al. [16], DE (MJ kg^−1^ DM) = 14.2 − 0.205 Acid Detergent Fibre (ADF) + 0.218 Ether Extract (EE) + 0.057 Crude Protein (CP) (*R*^2^ = 0.965, Relative Standard Deviation (RSD) = 0.494).

After one week of adaptation to the new environment, the collection of the data started. Mortality was recorded daily. The rabbits were individually weighed at the beginning and at the end of the trial to calculate the daily weight gain (DWG). The amount of feed was also weighed at the beginning and at the end of the trial to calculate the feed intake (FI) per replicate. Thus, the feed conversion ratio (FCR) was calculated as daily feed intake/average daily weight gain per replicate.

### 2.2. Blood Analyses

At 94 days of age, one rabbit for replicate (nine per groups) was chosen to reduce the differences in weight at slaughter among the groups. The rabbits were weighed, and blood samples from the ear vein were collected in plastic tubes with and without potassium ethylene diamine tetra-acetic acid (EDTA). The first aliquot was centrifuged at 2000× g for 15 min and serum was used for determination of biochemical parameters by means of an automatic biochemical analyser (AUTOLAB, AMS Corporation, Rome, Italy) using reagents from Spinreact (Santa Coloma, Spain). The second aliquot (EDTA) was used for complete blood count (CBC), performed within 30 min from the collection. CBC was performed using a semi-automatic cell counter (Genius S, SEAC Radom Group, Calenzano, Italy). In addition, May-Grünwald-Giemsa-stained blood smears were evaluated by an optic microscope for cell morphology, presence of abnormal cells or evidence of platelet clumping. Finally, the neutrophil/lymphocyte ratio (N/L) was calculated.

### 2.3. Carcass and Meat Traits

After blood collection, the rabbits were moved to a specialized slaughterhouse and the carcass traits were evaluated following the World Rabbit Science Association recommendations [17]. The slaughtered rabbits were bled, and the full gastrointestinal tract, skin, distal part of legs and tail, kidneys, genitals, and urinary bladder were removed. The carcasses were weighed and then chilled at 4 °C for 24 h in a ventilated room. After 24 h chilling, the carcasses were weighed again to obtain the chilled carcass (CC) weight, then head, liver, heart, the lungs + oesophagus + trachea + thymus gland package, and kidneys, were removed to obtain the reference carcass (RC). From the RC, hind legs (HL), Longissimus thoracis et lumborum (LTL) muscle and the rest of the meat were separated from the bone and fat (inguinal, abdominal and interscapular depots). All the meat, the bones and the fat were weighed and the meat to bone ratio was calculated. With a portable instrument (Model HI 9025; Hanna Instruments, Woonsocket, RI, USA), equipped with an electrode (FC 230C; Hanna Instruments), the value of pH was measured in the Biceps femoris (BF) and Longissimus lumborum (LL) muscles, 1 and 24 h after slaughtering. Crude protein, crude lipids, and ash contents of the Longissimus dorsi meat were determined by using 981.10, 991.36, and 920.153 of the AOAC [18] methods, respectively.

### 2.4. Caecal Microbial Activity

The caecum of each rabbit was tied at both ends, separated by sterile instruments from the rest of the gastrointestinal tract, placed in tightly closed plastic bags and put in pre-warmed thermos. After the sampling, the material was transported in the shortest possible time (about 1 h) to the laboratory, where two quotes of the caecal content (each about 5 mL) were used for the short chain fatty acids (SCFA) determination. The samples were diluted with oxalic acid (1:1, v/v) and SCFA were analyzed by a gas chromatography method [19] (Thermo-Electron mod. 8000top, FUSED SILICA Gaschromatograph, ThermoElectron Corporation, Rodano, Milan, Italy) equipped with an OMEGAWAX 250 fused silica capillary column 30 m × 0.25 mm × 0.25 mm film thickness, flame ionisation detector (185 °C), carrier helium (1.7 mL/min) under isothermal condition (125 °C).

### 2.5. Statistical Analysis

The data were processed by a one-way ANOVA, using the PROC GLM of SAS [20] according to the following model:

Yij = m + BSi + eij(1)

where Y is the single observation, m is the general mean, BS is the effect of the housing systems (i = F2F, Blind or Mirrors), e is the error. Comparison among means was performed by Tukey’s test [20] at *p* < 0.05. P values between 0.05 and 0.10 has been considered as “tendency”. For feed intake and feed conversion ratio, the replicate has been considered the experimental unit.

## 3. Results

No mortality was recorded along the trial and all the animals appeared healthy.

The Table 2 shows the effect of raising system on growth performance of the rabbits. The final weight and DWG were higher in F2F and Mirrors than in the Blind group (*p* < 0.05), while the FCR was more favorable in Mirrors than in Blind group (*p* < 0.01). The feed intake tended (*p* < 0.10) to be higher in the F2F than in the other groups.

Table 3 and Table 4 show the effect of the housing systems on carcass traits of rabbits at 1 h and 24 h after slaughter, respectively. Rabbits from blind groups tended to be lighter than those of the other groups. The percentage of the skin on live weight was lower in the blind than in the other groups (*p* < 0.01), while the percentage of empty gastro-intestinal tract (GIT) tended (*p* < 0.10) to be lower in the mirrors than in the other two groups. The percentage of the urogenital tract on the body weight was higher (*p* < 0.05) in the F2F than in the blind group. The gross dressing out (including GIT content) was higher (*p* < 0.05) in the mirrors than in the blind group, while, when the content of the gastro-intestinal tract was subtracted (net dressing out), the rabbits of the mirror group resulted in a higher (*p* < 0.05) carcass yield compared to both the blind and F2F groups.

The same tendency of gross and net dressing out was recorded for the chilled carcass (*p* < 0.05). Considering the inner organs, the only lungs + heart showed a higher percentage (*p* < 0.05) in F2F than in the other groups.

The Table 5 shows the effect of the raising system on the percentage of meat, bone and fat and on the Meat to Bone ratio, calculated on whole carcass. The percentage of meat was higher (*p* < 0.01) in F2F than in Mirrors group.

The proximate composition of meat was not affected by the rearing systems (Table 6).

The Table 7 shows the haematological traits of the rabbits raised under the different housing systems. The blind group had a higher (*p* < 0.05) value of red blood cells than the F2F, whilst the white blood cells tended (*p* < 0.10) to be lower in the mirrors group. The F2F group had the highest (*p* < 0.01) number of lymphocytes, while the mirrors group tended to have a higher number of platelets (*p* < 0.10) than the other groups. The N/L ratio the mean corpuscular volume (MCV) and mean corpuscular haemoglobin (MCH) values were lower in the blind than in the other groups (*p* < 0.01).

The Table 8 showed the effects of housing systems on serum biochemistry of rabbits. The F2F group had the lowest levels of glucose (*p* < 0.01) and the highest of urea (*p* < 0.05), CPK and LDH (*p* < 0.01). The blind had a serum total protein content higher (*p* < 0.01) than the mirrors group, while the globulin was higher in rabbits from F2F than mirrors group. The mirrors group had a higher level of cholesterol (*p* < 0.01) than the blind group and showed the lowest value of triglycerides, followed by the F2F and blind groups (*p* < 0.05).

The Table 9 shows the effect of the housing systems on the production of short-chain fatty acids in the caecum of the rabbits. The blind group showed the highest production of total SCFA, acetate (*p* < 0.05) and propionate (*p* < 0.01). The rabbits from the blind group also showed higher (*p* < 0.05) butyrate production than the mirrors group while the amount of isovaleric acid in F2F group was higher (*p* < 0.01) than in the mirrors group.

When the SCFA were expressed as a percentage of the total production, the mirrors group had the highest proportion of acetate (*p* < 0.01) and the lowest of propionate (*p* < 0.05). The blind group showed a proportion of butyrate higher than the mirrors group; the F2F group had the highest proportion of isovaleric acid (*p* < 0.01) and valeric acid in F2F group was higher than in blind group.

## 4. Discussion

Even if several studies showed a positive effect of mirrors on rabbit welfare [12,13,21], the use of this enrichment has been limited to the laboratory rabbits, probably because of the high cost of the application of the mirrors in the cages. Consequently, very little evidence is available in the literature on the effect of mirrors on rabbit growth performance [14]. Standing to our knowledge, no researches are available on the use of mirrors on free-range rabbits. In this case, the application of mirrors was easy due to adequate amount of available space. In addition, nowadays there is a large availability of more economic and safe materials that can be sanitized at the end of each production cycle further reducing the using costs.

Among the three tested, it seems that the rabbits from the Blind group, that have a real contact only with other two conspecifics and not simulated contact, showed a lower growth rate compared to the rabbits of the other groups. In addition, some haematological traits (i.e., MCV and MCH) were modified compared to the other groups, even if they fell in the physiological range for rabbit reported by Šimek et al. [22]. The lower percentage of the urogenital tract in Blind compared to F2F rabbits can be explained considering the lower live weight at slaughter of the first group. In fact, Garcia-Tomas et al. [23] reported a positive correlation between live and urogenital tract weights in male rabbits from 4 to 33 weeks of age, showing that live weight mainly affected the testis weight. The lower weight at slaughter can also be responsible of the lower percentage of the skin in the Blind group. The lower values of MCV and MCH was an expected results but, since no further analysis were made, we can just hypothesize that since rabbits from Blind group had a not different feed intake but a lower growth rate compared to the other groups, this could be suggestive of a poor feed utilization that also could have been reduced the absorption of iron. Indeed, the number of red blood cells was higher in the blind than in the F2F group while the haemoglobin concentration was similar among the groups. It is known that iron deficiency may be present despite a normal haemoglobin and full blood count: symptoms which may be prolonged and debilitating, should raise a clinical suspicion on iron deficiency even if full blood count is normal [24]. Indeed, the lifetime of rabbits bred for meat is probably too short to reach an anaemic state. Further, the RDW values were not different among the groups and this indicates that the volume of red cells was homogeneous inside each group: thus, the factors determining lower MCV act continuously [25]. However, more specific analysis should be needed to explain these effects on red blood cell parameters. Interestingly, the N/L ratio was significantly higher in the blind group, while no differences were detected for F2F and mirrors groups. This may suggest a higher level of cortisol in the blind group, thus, a low degree of stress in the other two groups. This ratio, as read from standard blood smears made before and after a stressful event, is positively related to the magnitude of the stressor and to the circulating glucocorticoids [26]. The implication is that increases in N/L ratios are observed in response to stressors of an animal’s environment [27]. On these bases, the absence of differences between F2F and mirrors should suggest that mirrors may simulate the F2F condition leading to a lower level of stress.

Jones et al. [12] observed that rabbits did not respond to mirror images as if they were conspecifics, but the enrichment was able to improve the welfare of the animals. The rabbits of the blind group showed a lower AST value than the other two groups and this may be suggestive of a better hepatic function. On the other hand, AST is a mitochondrial enzyme considered a less specific indicator of liver function than other enzymes since it can also be found in many peripheral tissues (i.e., muscles) and hence it has a very wide variability [28]. Moreover, ALT showed no differences among the groups, and, being considered a more specific indicator of liver function since localized on the cell membrane of the bile ducts [29], an effect on liver function should be excluded.

Looking at the fermentation patterns in the caecal content, the higher amount of total SCFA in Blind group indicated a higher fermentation in the caecum. This could be due to an effective higher fermentative activity of microbiota or to a higher availability of nutrients escaped from digestion and available for caecal fermentations. F2F and mirrors groups showed a similar amount of total and singular SCFA (except for isovaleric acid), but when the SCFA are expressed as percentage of the total produced amount, the F2F group is more similar to the blind. The proportion of the main SCFA for all the groups falls in the range indicated by Gidenne [30] who indicated that per 100 moles of SCFA produced, 60–80 are acetate, 8–20 are butyrate, and 3–10 are propionate. However, the mirrors group showed a higher proportion of acetate and lower of propionate and butyrate. It is known [31] that acetic acid production originates from the fermentation of cellulolytic bacteria, while butyrate and propionate are from non-structural carbohydrates fermentations. Our results seem to indicate that rabbits from the mirrors group were more able to ferment structural than non-structural carbohydrates. However, the propionate to butyrate ratio resulted not different among the groups. The butyrate is considered the main enterocytes energy source [32], due to the relatively high affinity of the colonocytes for butyrate and thus and it is generally considered necessary for a proper development of the GIT-associated lymphoid tissue [33]. However, it has been shown [34] that under physiological conditions, with a relative high concentration of acetate compared with butyrate, acetate is at least as important as butyrate for the energy supply in colonocytes of humans and rats. It is well known that the improvement of intestinal microbiota growth and development has an important impact on animals’ health and welfare [35]. Isovaleric and valeric acids are included among branched chain fatty acids (BCFA) and are produced from the degradation of the aminoacids leucine and proline in the cecum [36]. As the groups were fed the same diets, the differences in BCFA production suggest a higher degradation activity of caecal microbiota on the protein, according to Bovera et al. [32,37]. Thus, it seems that the housing systems can affect the activity of caecal microbiota, but further analyses are needed to better understand the exact kind of modification.

The higher live weight of rabbits from the mirrors group in comparison to the blind group is in line with the findings of Reddi et al. [14] who observed a higher growth rate of rabbits raised individually but with mirrors compared to the control group. The rabbits from F2F and mirrors groups reached a similar live weight at the end of the trial and also had similar FCR, but the net dressing out was higher in the mirrors group, due to a higher amount of digesta in the gastro-intestinal tract at the slaughter (426.8 g vs. 253.9 and 237.3 for mirrors, F2F and blind groups, respectively). The rabbits from F2F group showed higher levels of CPK and LDH and low values of blood glucose than those of the other groups. This is probably due to the higher locomotion activity of these rabbits as described in the first part of this research [38]. In fact, rabbits from F2F groups spent around 37.9 m/d in locomotion activity, while those of the other groups only 25.1 (blind group) or 28.5 (mirrors) (*p* < 0.05). In addition, F2F rabbits spent less time to rest 135.6 m/day vs. 196.2 and 173.0 of blind and mirrors group, respectively. The higher muscular activity increased the values of both LDH and CPK enzymes, and such results may also explain the higher AST levels respect to the blind group, thus confirming that differences in AST should have muscular rather than hepatic origin. In addition, the high energy expenditure along the day, reduced the amount of glucose in the serum of the F2F group rabbits. The tendency to a higher feed intake recorded for F2F rabbits could be ascribed to the increase in the energy requirements due to the higher locomotion activity [8]. In addition, the tendency to a higher feed intake of the F2F rabbits could be also responsible for the tendency to a higher percentage of their empty gastro-intestinal tract. Finally, the increased muscular exercise was probably responsible of the increased muscular mass in rabbits from F2F group.

## 5. Conclusions

In conclusion, when the rabbits were raised in groups consisting of three animals and isolated from other conspecifics, they showed a lower growth rate and lower values of MCV and MCH compared to the other groups. These negative aspects disappeared when mirrors were inserted in the raising area and, in addition, the use of mirrors increased the net dressing out percentages in comparison to the other groups. The use of mirrors can lead to an enhancement of growth performance and carcass traits by lowering the rabbit’s locomotion activity and thus the energy expenditure compared to the other tested systems.

## Figures and Tables

**Table 1:** Chemical characteristics of the diet.

Chemical Composition	Item
Crude protein, % as feed	15.6
Ether extract, % as feed	3.6
Ash, % as feed	7.4
Acid Detergent Fibre, % as feed	19.4
Ca, % as feed	0.74
P, % as feed	0.53
Na, % as feed	0.23
Lysine, % as feed	0.66
Methionine, % as feed	0.22
Digestible Energy, MJ/kg	11.9

Ingredients: dehulled sunflower seeds f.e., wheat bran, beet pulp, dehydrated alfalfa meal, hop, grapeseed f.e., barley, wheat flour middling, molasses, soybean oil, CaCo_3_, NaCl, palm oil, MgO. MinVit integration: Vit A 15,000 UI, Vit D 1,500 UI, Vit E 50 mg. MnO 50 mg, ZnO 70 mg, CaCo 0.13 mg, CuSO_4_ 10 mg, FeSO_4_ 150 mg, Ca (IO_3_)_2_ 3 mg, Na_2_SeO_3_ 0.10 mg, Robenidine chloride 66 mg.

**Table 2:** Effect of raising system on growth performance of rabbits.

Rabbits’ Performance	Initial Weight (g)	Final Weight (g)	DWG (g/d)	FI (g/d)	FCR
Face to Face	1421.5	2496.9 ^a^	25.01 ^a^	132.0	5.36 ^AB^
Blind	1419.8	2334.6 ^b^	21.28 ^b^	125.2	5.88 ^A^
Mirrors	1426.6	2490.5 ^a^	24.74 ^a^	122.1	4.97 ^B^
RMSE	79.76	85.91	2.23	9.81	0.35
*p-*Value	0.9841	0.0423	0.0461	0.0929	0.0002

DWG: daily weight gain; FI: feed intake; FCR: feed conversion ratio. RMSE: root mean square error; a, b: *p* < 0.05; A, B: *p* < 0.01.

**Table 3:** Effect of housing systems on “hot” carcass traits (within 1 h from slaughter).

Carcass’ Traits	Face to Face	Blind	Mirrors	RMSE	*p*-Value
Live weight, g	2420.0	2340.0	2410.7	428.8	0.0843
Skin, % LW	16.55 ^A^	14.76 ^B^	16.87 ^A^	0.79	0.0004
Full GIT, %LW	20.58	21.01	26.83	5.91	0.1396
Empty GIT, %LW	10.09	10.87	8.51	1.67	0.0879
Urogenital tract, %LW	0.2435 ^a^	0.1415 ^b^	0.1968 ^ab^	0.0649	0.0322
Spleen, % LW	0.079	0.078	0.071	0.0229	0.8021
Hot carcass, g	1372.5	1260.0	1420.0	326.3	0.6867
Gross dressing out, %	56.71 ^ab^	53.73 ^b^	58.90 ^a^	1.98	0.0352
Net dressing out, %	64.05 ^b^	63.05 ^b^	69.38 ^a^	1.61	0.0250
pHLD1h	6.75	6.63	6.75	0.2954	0.6969
pHBF1h	7.10	7.17	7.06	0.3387	0.8400

LW: live weight; pHLD1h: pH of the Longissimus dorsi muscle 1 h after slaughter; pHBF1h: pH of the Biceps femoris muscle 1 h after slaughter; GIT: gastro-intestinal tract; RMSE: root mean square error; a, b: *p* < 0.05; A, B: *p* < 0.01.

**Table 4:** Effect of housing systems on chilled carcass traits (after 24 h of refrigeration at 4 °C).

Carcass’ Traits	Face to Face	Blind	Mirrors	RMSE	*p*-Value
Chilled carcass, g	1357.9	1253.0	1402.0	322.3	0.7152
Gross dressing out, %	56.11 ^ab^	53.54 ^b^	58.16 ^a^	2.09	0.0184
Net dressing out, %	63.30 ^b^	62.57 ^b^	68.61 ^a^	2.82	0.0273
RC, g	1092.3	1175.7	1168.8	309.0	0.8521
Liver, % CC	5.07	6.17	4.84	1.45	0.5321
Kidney, % CC	1.36	1.38	1.05	0.43	0.4123
Lungs + heart, % CC	2.50 ^a^	187 ^b^	1.83 ^b^	0.20	0.0324
Head, % CC	11.44	11.22	10.10	2.11	0.1196
Carcass length, cm	43.50	42.33	44.17	1.54	0.1417
Carcass circumference	18.75	16.67	17.50	1.88	0.1445
pHLD24h	5.86	5.93	5.82	0.17	0.5588
pHBF24h	6.04	6.00	5.95	0.1384	0.4387

RC: carcass of reference; pHLD24h: pH of the Longissimus dorsi muscle 24 h after slaughter; pHBF24h: pH of the Biceps femoris muscle 24 h after slaughter; RMSE: root mean square error; a, b: *p* < 0.05.

**Table 5:** Effect of the housing systems on the percentage of meat, bone, fat and on the meat to bone ratio (M:B) calculated on the whole carcass.

Carcass’ Constituents	Face to Face	Blind	Mirrors	RMSE	*p*-Value
Meat, % RC	70.77 ^A^	69.52 ^AB^	68.61 ^B^	1.103	0.0070
Bone, % RC	28.06	28.93	27.97	3.13	0.2312
Fat, % RC	0.45	0.86	2.57	1.92	0.1259
M:B	2.52	2.40	2.45	0.13	0.1836

RC: carcass of reference; RMSE: root mean square error; A, B: *p* < 0.01.

**Table 6:** Effect of the housing systems on the percentage of crude protein, crude lipids and ash (in g/100 g dry matter) of the Longissimus lumborum.

Carcass’ Composition	Face to Face	Blind	Mirrors	RMSE	*p*-Value
Crude protein	90.88	90.34	92.08	1.711	0.486
Crude lipids	3.58	2.10	1.73	1.161	0.201
Ash	5.14	5.75	5.38	0.403	0.258

RMSE: root mean square error.

**Table 7:** Hematological traits of rabbits according to the housing systems.

Hematological Traits	Face to Face	Blind	Mirrors	RMSE	*p*-Value
Hematocrit, %	37.7	41.0	40.8	4.95	0.3416
Hemoglobin, g/dL	10.8	11.6	11.6	1.43	0.4681
RBC, 10^3^/mm^3^	5.75 ^b^	6.65 ^a^	6.26 ^ab^	0.69	0.0444
WBC, 10^3^/mm^3^	8.06	7.29	4.49	2.09	0.0890
Neutrophils, 10^3^/mm^3^	4.51	4.51	2.07	1.85	0.0911
Lymphocytes, 10^3^/mm^3^	3.25 ^A^	1.67 ^B^	1.36 ^B^	0.47	<0.0001
Eosinophils, 10^3^/mm^3^	0.23	0.55	0.05	0.46	0.1055
Monocities, 10^3^/mm^3^	0.40	0.66	0.70	0.602	0.5615
Basophils, 10^3^/mm^3^	0.12	0.01	0.02	0.014	0.1708
N/L	1.39 ^B^	2.71 ^A^	1.51 ^B^	0.1423	0.0093
RDW, %	15.35	15.83	14.81	1.08	0.9471
Platelets, 10^3^/μL	240.0	190.1	491.6	106.4	0.0878
MPV, fL	5.72	4.98	5.72	0.69	0.1185
MCV, fL	65.6 ^A^	61.6 ^B^	65.1 ^A^	2.10	0.0017
MCH, pg	18.9 ^A^	17.4 ^B^	18.5 ^A^	0.71	0.0016
MCHC, g/dL	28.8	28.4	28.5	0.52	0.5816

RBC: red blood cells; WBC: white blood cells; RDW: red cell distribution width; MPV: Mean platelet volume; MCV: mean corpuscular volume; MCH: mean corpuscular hemoglobin; MCHC: mean corpuscular hemoglobin concentration; N/L: neutrophil/lymphocyte ratio; RMSE: root mean square error; a, b: *p* < 0.05; A, B: *p* < 0.01.

**Table 8:** Serum biochemistry of rabbits according to the housing systems.

Serum Biochemistry	Face to Face	Blind	Mirrors	RMSE	*p*-Value
Glucose, mg/dL	112.5 ^B^	132.5 ^A^	135.4 ^A^	11.5	0.0007
Total Protein, g/dL	6.63 ^AB^	7.10 ^A^	6.23 ^B^	0.40	0.0013
Albumin, g/dL	3.51	3.37	3.40	0.15	0.1383
Globulin, g/dL	3.33 ^a^	3.02 ^ab^	2.92 ^b^	0.35	0.0463
A/G	1.06	1.13	1.16	0.13	0.2268
Cholesterol mg/dL	58.6 ^AB^	49.7 ^B^	64.0 ^A^	6.87	0.0257
Triglycerides mg/dL	88.4 ^b^	99.0 ^a^	69.0 ^c^	8.56	0.0298
Urea, mg/dL	59.2 ^a^	44.0 ^b^	37.0 ^b^	9.76	0.0471
Creatinine, mg/dL	1.41 ^b^	1.35 ^b^	1.63 ^a^	0.14	0.0380
ALT, U/L	80.2	72.3	71.0	15.72	0.3994
AST, U/L	62.0 ^A^	36.0 ^B^	51.8 ^A^	11.53	0.0010
CPK, UI/L	2356.3 ^A^	1296.8 ^B^	1189.6 ^B^	540.9	0.0005
LDH, UI/L	825.8 ^A^	367.7 ^B^	472.6 ^B^	203.1	0.0005

A/G: albumin to globulin ratio; ALT: alanine aminotransferase; AST: aspartate aminotransferase; CPK: creatine phosphokinase; LDH: lactate dehydrogenase; RMSE: root mean square error; a, b, c: *p* < 0.05; A, B: *p* < 0.01.

**Table 9:** Effect of housing systems on short-chain fatty acid (SCFA) production in rabbit’s caecum.

SCFA	Face to Face	Blind	Mirrors	RMSE	*p*-Value
Mmol/L					
Acetate	24.3 ^b^	33.2 ^a^	24.1 ^b^	2.83	0.0230
Propionate	2.27 ^B^	3.54 ^A^	1.86 ^B^	0.32	0.0057
Butyrate	2.81 ^ab^	4.33 ^a^	2.22 ^b^	0.38	0.0235
Isovaleric acid	0.35 ^A^	0.23 ^AB^	0.15 ^B^	0.02	0.0046
Valeric acid	2.55	2.48	2.38	0.43	0.6791
C_3_/C_4_	0.81	0.82	0.84	0.07	0.8521
Total SCFA	32.5 ^b^	43.9 ^a^	30.9 ^b^	3.77	0.0114
% total SCFA					
Acetate	74.5 ^B^	75.3 ^B^	78.1 ^A^	1.77	0.0005
Propionate	7.21 ^a^	7.76 ^a^	6.07 ^b^	0.37	0.0174
Butyrate	7.97 ^ab^	9.80 ^a^	7.13 ^b^	0.81	0.0223
Isovaleric acid	1.19 ^A^	0.54 ^B^	0.49 ^B^	0.39	0.0011
Valeric acid	8.24 ^a^	6.05 ^b^	7.69 ^ab^	0.55	0.0367

C_3_/C_4_: propionate to butyrate ratio; RMSE: root mean square error; a, b: *p* < 0.05; A, B: *p* < 0.01.
